# Comparison of Ventilation Strategies Across the Perioperative Period in Patients Undergoing General Anesthesia: A Narrative Review

**DOI:** 10.7759/cureus.77728

**Published:** 2025-01-20

**Authors:** Taysir M Alnsour, Mohammed Ahmad Altawili, Shaima Mohammed A Alghuraybi, Joud Essa Alshammari, Anwar Ghudair T Alanazi, Menwer Ghudair T Alanazi, Abdirazak Ahmed Ali Nur, Manar A Alharbi, Albandari Saad Alanazi

**Affiliations:** 1 Anaesthesiology, King Fahad Specialist Hospital, Tabuk, SAU; 2 General Practice, King Fahad Specialist Hospital, Tabuk, SAU; 3 Anesthesiology, King Saud University Medical City, Riyadh, SAU; 4 General Practice, King Abdulaziz University, Jeddah, SAU; 5 Medicine, King Khalid General Hospital, Hafar Al Batin, SAU; 6 Anesthesiology, Majmaah University Faculty of Medicine, Al Majma'ah, SAU; 7 General Practice, International University of Africa, Khartoum, SDN; 8 General Practice, Umm Al-Qura University, Jeddah, SAU; 9 Anesthesiology, University of Tabuk, Tabuk, SAU

**Keywords:** anesthesia, general anesthesia, oxygenation, review, strategies, ventilation

## Abstract

General anesthesia is a critical component of surgical procedures, requiring effective ventilation strategies to ensure adequate oxygenation and prevent complications. This narrative review aims to compare various ventilation techniques used during general anesthesia, focusing on their physiological foundations, clinical applications, and outcomes. Traditional methods, such as high tidal volume ventilation, have evolved into more sophisticated approaches, including protective lung ventilation, which are particularly beneficial for high-risk patients with respiratory comorbidities. The review highlights that protective lung ventilation, characterized by lower tidal volumes and optimal positive end-expiratory pressure, is associated with improved oxygenation, reduced incidence of post-operative pulmonary complications, and enhanced overall recovery. Despite the advantages of personalized ventilation approaches, current evidence remains limited by small sample sizes and variability in study designs. This underscores the need for larger, randomized controlled trials to establish definitive guidelines. Future research should also explore emerging technologies to optimize the real-time management of ventilation parameters. The findings emphasize the importance of individualized ventilation strategies in clinical practice to improve patient outcomes and inform policy development. By advancing our understanding of ventilation techniques, this review aims to contribute to safer anesthesia practices and enhance recovery in surgical patients.

## Introduction and background

General anesthesia (GA) is a medical state induced to facilitate surgical procedures, ensuring the patient remains unconscious, immobile, and free from pain. The administration of GA involves a multifaceted approach, with ventilation strategies playing a pivotal role in the overall management of anesthesia. Adequate ventilation is crucial not only for the maintenance of normal physiological functions but also for the prevention of complications during and after surgery [1].

The primary goal of ventilation during GA is to ensure sufficient oxygen delivery and carbon dioxide removal [2]. Historically, traditional methods such as manual ventilation and the use of endotracheal tubes have been standard practices. However, advancements in anesthesia technology have led to the development of various ventilation strategies, including volume-controlled mechanical ventilation, pressure-controlled ventilation (PCV), and adaptive support ventilation [3]. Each of these methods presents unique advantages and limitations that can impact patient outcomes, particularly in high-risk populations such as those with respiratory comorbidities or in prolonged surgical procedures [4,5].

**Figure 1:**
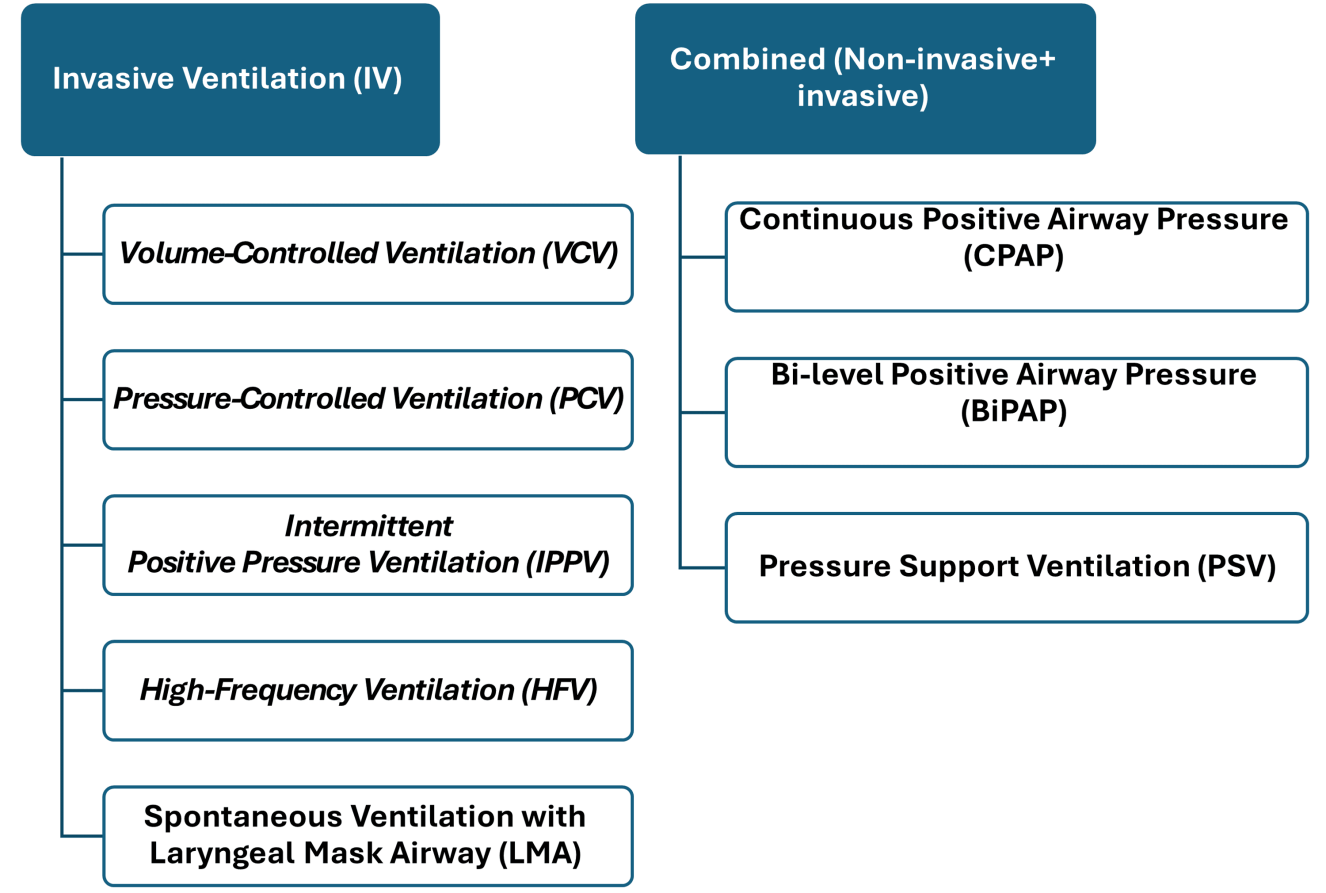
Different strategies for invasive and non-invasive mechanical ventilation. This figure is original and created by the authors. NB: BiPAP and CPAP are used during intubation if the effects of muscle relaxants have worn off

The importance of selecting an appropriate ventilation strategy cannot be overstated (Figure 1). Studies have shown that inadequate ventilation can lead to hypoxemia, hypercapnia, and even respiratory failure, resulting in increased morbidity and mortality rates [6]. Furthermore, the choice of ventilation strategy may influence intraoperative hemodynamics, the incidence of PPCs, and the duration of recovery [7]. For instance, studies have demonstrated that patients who receive protective lung ventilation (PLV) strategies, which are characterized by lower tidal volumes and optimal positive end-expiratory pressure (PEEP), exhibit improved respiratory outcomes compared to those receiving conventional ventilation techniques [8].

The principles of lung protection are increasingly recognized as integral to minimizing ventilator-induced lung injury (VILI), a phenomenon that can compromise recovery and increase morbidity in surgical patients [9]. Recent evidence suggests that personalized ventilation approaches tailored to individual patient needs and surgical contexts can optimize respiratory mechanics and improve overall outcomes [10].

Despite the clear significance of effective ventilation in general anesthesia, there remains a lack of consensus regarding the optimal strategy for various clinical scenarios. By comprehensively reviewing existing literature, this review aims to provide a nuanced understanding of various ventilation strategies used during general anesthesia. It seeks to compare these techniques, highlighting their physiological underpinnings, clinical applications, and associated outcomes, and ultimately offers recommendations for clinical practice based on synthesizing recent findings.

## Review

Methodology

In October 2024, a comprehensive search was conducted across PubMed, Scopus, and Web of Science using keywords such as "Ventilation", "General anesthesia", and Medical Subject Headings (MeSH). Additionally, key references were identified from the bibliographies of relevant studies. To narrow the focus to specific study types, filters were applied for clinical trials, meta-analyses, and systematic reviews, covering October 2014 to October 2024.

Definition of ventilation

It is the process of gas exchange in the lung alveoli that provides sufficient oxygen for tissue perfusion. It is mainly conducted by integrating multiple organ systems, which are mainly the lung circulation and the extended capillaries to the alveoli, which represent the cardiovascular system's involvement, the diaphragm and intercostal muscles, which represent the musculoskeletal system, and surely the respiratory system with its alveoli [11].

Types of ventilation (Invasive vs. Non-invasive)

The difference between invasive and non-invasive mechanical ventilation (NIMV) is primarily based on the need for a patient's endotracheal intubation to deliver oxygen and anesthetic gases. In cases of invasive ventilation, an endotracheal tube is inserted to deliver the required oxygen and eliminate CO_2_. It could be driven by a machine (mechanical ventilation) or controlled by a healthcare provider manually (manual ventilation) [12]. On the other hand, in NIMV, the air doesn't need to be delivered via any form of invasion; however, the air is pumped via an oral or nasal mask. Many forms of NIMV strategies exist: Continuous positive airway pressure (CPAP), bilevel positive airway pressure (BiPAP), and NIMV with pressure support ventilation (PSV) [13]. Choosing the optimum method of ventilation depends on the patient's status and requirements to compensate for the oxygen deprivation.

Physiological principles of ventilation

A complex integrated process between muscular, neural, and respiratory elements is implemented to provide the body's oxygen requirements by ventilation. The pressure gradient is the basis of ventilation. The diaphragm starts by contracting and increasing the volume of the chest cavity. This creates a negative pressure gradient, causing air to flow into the lungs in inhalation. Then the diaphragm relaxes, reducing the volume of the chest cavity and expelling air in exaltation. The competency of ventilation depends on several factors: lung compliance for airflow exchange, airway resistance, involved respiratory muscle strength, and neural control of the depth of breathing [14]. Age, health problems, and the surrounding environment also affect the ventilation efficiency [15]. However, the respiratory system is essential for preserving the acid-base equilibrium, too. The lungs are responsible for disposing of carbon dioxide. Tidal volume and respiratory rate (minute ventilation) are modified to keep blood carbon dioxide levels within acceptable ranges [16].

Patient-specific considerations

Age and Comorbidities

The choice of ventilation strategy for patients undergoing GA must be tailored to individual patient characteristics, especially age. This significantly influences respiratory mechanics, hemodynamic stability, and overall surgical outcomes. In elderly patients, for instance, physiological changes such as decreased lung compliance, reduced respiratory muscle strength, and altered gas exchange dynamics necessitate a careful approach to ventilation management [17]. Studies have shown that older adults are at a higher risk of PPCs, underscoring the importance of employing lung-protective ventilation strategies that minimize the risk of VILI [18].

Comorbidities, particularly chronic obstructive pulmonary disease (COPD), obesity, and obstructive sleep apnea (OSA), further complicate the selection of ventilation methods. Patients with COPD often require specific ventilatory adjustments to accommodate increased airway resistance and dynamic hyperinflation [19]. Similarly, obese patients present unique challenges due to reduced functional residual capacity and increased work of breathing, making strategies such as high-frequency oscillatory ventilation or enhanced PEEP particularly beneficial in optimizing gas exchange [20].

Type of Surgery

Different surgical procedures present unique physiological challenges and patient-specific factors that necessitate tailored approaches to ventilation. In thoracic procedures, such as lobectomies or cardiac surgeries, one of the primary considerations is the impact on pulmonary function. Strategies like one-lung ventilation (OLV) are often employed to facilitate surgical exposure and minimize lung injury. Studies have shown that OLV can improve surgical outcomes but requires careful monitoring of hemodynamics and oxygenation [21]. However, research indicates that using low tidal volume ventilation during OLV has not been linked to a reduction in PPCs, and no independent association was found between a low tidal volume lung-protective ventilation regimen and a composite of these complications [22,23].

In abdominal surgeries, such as laparoscopic procedures, the effects of pneumoperitoneum on respiratory mechanics must be considered. The increased intra-abdominal pressure can impair diaphragmatic movement and lung compliance, necessitating adjustments in ventilatory strategies [24]. Studies indicate that high-frequency oscillatory ventilation may be beneficial in this context, helping to maintain adequate gas exchange while mitigating the risks of barotrauma [25].

For orthopedic surgeries, especially those involving prolonged anesthesia and positioning, strategies such as PEEP can help prevent atelectasis and maintain oxygenation. The choice of PEEP levels may vary based on the patient's lung function and the anticipated duration of surgery [26].

Emergency vs. Elective Surgery

**Figure 2:**
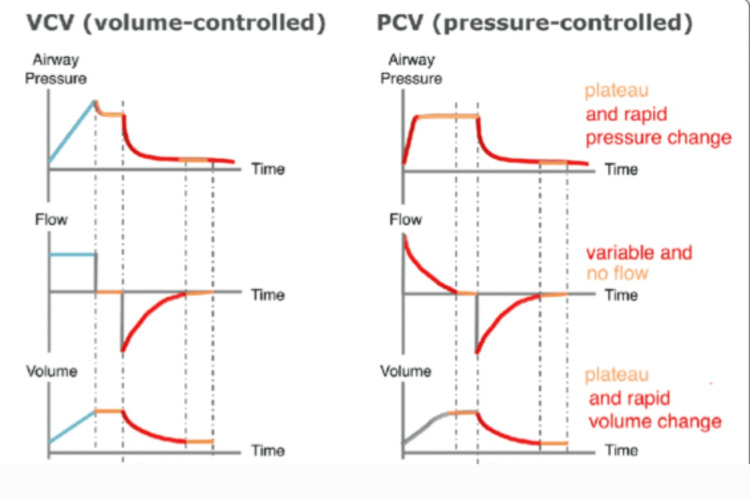
Volume-controlled ventilation vs. pressure-controlled ventilation Available from: https://www.researchgate.net/figure/Flow-controlled-ventilation-FCV-compared-to-volume-controlled-ventilation-VCV-and_fig1_362962961 (Open access)

In elective surgeries, where patient stability can be effectively managed, controlled ventilation strategies-particularly volume-controlled ventilation-are typically favored to ensure adequate gas exchange and reduce the risk of respiratory complications. A systematic review and meta-analysis indicate that volume-targeting and PCV modes can enhance airway dynamics in both two-lung and OLV scenarios. Additionally, these modes have improved oxygenation during OLV in adults undergoing elective procedures (Figure 2) [27].

In contrast, emergency surgeries often necessitate rapid intubation and may utilize more flexible approaches, such as PCV, to address the urgent nature of the procedure and the patient's potentially unstable condition [28]. Research indicates that a lung-protective ventilation strategy is preferred for patients requiring mechanical ventilation in prehospital and emergency settings. This can be achieved by administering low tidal volumes while concurrently titrating both FiO2 and PEEP to ensure adequate oxygenation [29].

Invasive mechanical ventilation

IMV is typically administered through an endotracheal tube, necessitates tracheal access, and is the most common reason for intensive care unit (ICU) admissions (Table 1). IMV is a critical intervention used during general anesthesia, providing essential respiratory support to patients unable to maintain adequate ventilation. IMV, typically administered through an endotracheal tube, necessitates tracheal access and is the most common reason for ICU admissions [30]. One of the primary advantages of IMV is its ability to deliver precise ventilation parameters, allowing for optimal gas exchange and oxygenation, particularly in patients with compromised pulmonary function. IMV can be tailored to meet individual patient needs, enabling the use of lung-protective strategies that minimize VILI. Furthermore, IMV is often essential for prolonged procedures or in cases where rapid changes in ventilation are required [31]. However, IMV also carries significant risks. The invasive nature can lead to complications such as airway trauma, pneumothorax, and increased incidence of ventilator-associated pneumonia (VAP) [32]. Additionally, the need for sedation to tolerate intubation may complicate recovery and prolong post-operative ventilation [33]. Lastly, IMV requires continuous monitoring and management, placing a strain on healthcare resources, particularly in emergency or high-demand settings [34].

**Table 1:** Comparison between different combined mechanical ventilation strategies (Invasive and non-invasive)

Ventilation Strategy	Volume	Pressure	Indication	Advantage	Disadvantage
Continuous Positive Airway Pressure (CPAP)	Variable	Continuous positive pressure	Postoperative care, sleep apnea	Enhances oxygenation	Risk of lung injury
Bi-level Positive Airway Pressure (BiPAP)	Variable	Two pressure levels	Chronic obstructive pulmonary disease (COPD)	Enhances oxygenation	Risk of discomfort
Pressure Support Ventilation (PSV)	Variable	Additional pressure support	Weaning from mechanical ventilation	Reduces work of breathing	Requires patient effort

Pressure-Controlled Ventilation (PCV)

In this type of ventilation, constant pressure is delivered to the patient's lung during the whole inspiratory phase. According to the patient's lung compliance and capacity, the volume of air received by the ventilator is determined [37]. The constant pressure applied to the lungs would increase the mean airway pressure, which subsequently would lead to increased alveolar recruitment and enhanced tissue and blood oxygenation [38]. Contrary to VCV, PCV reduces the risk of developing VILI, as it limits the PIP to the patient's limits. However, the high setting of the applied air pressure could increase the risk of barotrauma, and a low-pressure setting could cause hypoventilation [38]. Other advantages of this technique are that, based on the fact the ventilator delivers the air only through the inspiration phase, the synchroneity between the patient and the machine brings comfort to the patients. The constant applied pressure also compensates for any leak in the airway circuit, stabilizing the patient's oxygenation. The drawbacks of this technique are that, as lung compliance could change during the ventilation, close monitoring and constant adaptation of the applied pressure to the lungs should be done to avoid any complications. Also, if the provided airflow is not matched, the patient's effort in the inspiration phase would increase the load on the accessory muscle work [39]. 

Intermittent Positive Pressure Ventilation (IPPV)

In this mode of ventilation, the ventilator provides pressurized, positive airflow in a constant, intermittent pattern that would maintain a potent airway for the patient's spontaneous breathing between the ventilator's breathing intervals [40]. The mode provides sustained and controlled ventilation to the patients, which would be suitable with patients requiring specific respiratory needs as it controls the respiratory rate and tidal volume [41]. However, patients with this mode of ventilation may show discomfort as a result of dyssynchrony. Also, patients with changed lung compliance have an increased risk of barotrauma [42].

High-Frequency Ventilation (HFV)

HFV employs very high respiratory rates (over 60 breaths per minute) with small tidal volumes (often less than the anatomical dead space) delivered at supra-physiological frequencies [43]. There is growing interest in minimizing movement secondary to ventilation, a concept known as motion management, for various ablation techniques. HFV appears to be a promising ventilatory strategy during airway or tracheal surgeries, as well as in the prevention of barotrauma in patients with compromised lung compliance. It is also used in the management of bronchopleural fistula, neonatal and pediatric anesthesia, severe hypoxemia or hypercapnia unresponsive to conventional ventilation, thoracic trauma cases, and procedures involving one-lung ventilation to optimize oxygenation. However, further studies are needed to confirm its benefits and assess potential risks in this context [25].

Spontaneous Ventilation with Laryngeal Mask Airway

LMA is considered a type of invasive method of applying ventilation. It is less invasive compared to endotracheal tubes. It allows for effective ventilation with minimal instrumentation of the airway, which is why it is often used in surgeries requiring GA and as a rescue device for difficult airways [44].

Modes of ventilation

Pressure-Regulated Volume Control (PRVC)

In this mode of ventilation, both PVC and VCV were integrated. It is monitored by providing a set tidal volume with each breath. The ventilator in this mode is self-adjusted as it observes the previous breaths, and if noticed to be associated with decreased tidal volume, it increases the pressure to compensate and provide the optimum air pressure to maintain good oxygenation [45]. The sustained pressure would optimize the number of alveoli recruited, the patient's comfort, and the harmony between the machine and the patient's spontaneous ventilation [39]. This would also protect the lungs from any ventilation-induced lung injury, solve the need for close monitoring in PVC, and provide an excellent option for cases needing precise ventilation, like acute respiratory distress syndrome (ARDS) [46]. Despite its many advantages, the complexity of the ventilator faced in its setting raises the chances of the wrong setting of the machine or misinterpretation of its alarms. Also, the automatic nature of this mode may not be able to cover the variability of the target tidal volume needed in cases of asthma and COPD. It was also noticed that when lung compliance and alveolar recruitment were achieved, the ventilator could also decrease the provided tidal volume so low that the patients could go through hypoventilation [47].

Protective Lung Ventilation (PLV)

This mode provides lower tidal volumes for patients and higher levels of PEEP. According to titration of FiO₂ and PEEP during protective lung ventilation (PLV). It aims to balance oxygenation and lung protection. FiO₂ is initially set high (e.g., 100%) during induction, then reduced to the lowest level, maintaining SpO₂ at 92%-96% or PaO₂ at 60-80 mmHg to avoid hyperoxia. PEEP is typically started at 5 cmH₂O and adjusted in small increments (e.g., 2 cmH₂O) to prevent atelectasis while minimizing overdistension, guided by oxygenation, lung compliance, and driving pressure (≤15 cmH₂O). Optimal titration combines FiO₂ and PEEP adjustments, often using protocols like FiO₂-PEEP tables, to achieve oxygenation goals while protecting against ventilator-induced lung injury [48]. This ventilation strategy would protect the patients from any incidences of overdistension and alveolar collapse [49]. A systematic review and meta-analysis conducted by Zhang et al. 2015 on patients getting PLV undergoing major surgeries showed that PLV significantly protected against acute lung injury (ALI) [50]. However, using low tidal volume can cause the patients to go through hypercapnia, not meeting the patient's ventilation needs. Patients have also reported some discomfort and increased breathing effort. Also, the PEEP could cause overdistention of the alveoli and impair gas exchange [51]. 

One-Lung Ventilation (OLV)

This mode of ventilation is conducted in surgeries by isolating one lung from the other so that patients can go through surgeries in one lung while keeping the other intact by using a double tube to ventilate one and keeping the other collapsed [52]. However, the collapsed lung may develop hypoxemia because of the shortage of oxygen intake in the body, which would affect the oxygen supply to the collapsed lung. Although some techniques would compensate for this shortage by increasing lung capacity, increasing alveolar recruitment, and decreasing vascular resistance in the collapsed lungs, this would compensate for possible hypoxemia [53].

Combined (Non-invasive and invasive mechanical ventilation strategies) 

NIMV has emerged as a valuable alternative to invasive methods in various clinical scenarios during general anesthesia (Table 2). Its primary advantage is minimizing airway trauma and complications associated with intubation while providing effective respiratory support. NIMV can help improve oxygenation and reduce the work of breathing, making it particularly useful during procedures that may exacerbate respiratory compromise. However, each one of these non-invasive strategies could be an invasive ventilation strategy when used during intubation if the effects of muscle relaxants have worn off.

NIMV is also indicated in the post-operative setting, particularly for patients undergoing thoracic or upper abdominal surgery, to mitigate the risk of respiratory complications and facilitate recovery [54].

Moreover, it serves as a valuable bridge for critically ill patients who may deteriorate rapidly, allowing for timely respiratory support without immediate intubation. A systematic review and meta-analysis of randomized control trials concluded that utilizing NIMV for weaning from mechanical ventilation reduces hospital mortality, lowers the incidence of VAP, and shortens ICU stays. This weaning strategy is particularly advantageous for patients with COPD [55].

Furthermore, NIMV has been suggested as a preventive measure to avoid reintubation (preventive NIMV) or as an emergency intervention for post-extubation respiratory failure (rescue NIMV). The early application of NIMV immediately after extubation has effectively prevented post-extubation acute respiratory failure (ARF). However, it has not demonstrated benefits in preventing reintubation for patients who develop ARF after extubation, with higher mortality rates observed compared to those receiving standard treatment. Therefore, while NIV should be considered a tool to prevent post-extubation ARF, further research is needed to identify which patient groups may benefit the most from its use in this setting [56,57].

NIMV has demonstrated considerable efficacy in surgical settings, particularly in reducing respiratory complications and facilitating recovery. Studies show that NIMV can improve oxygenation and decrease the need for invasive intubation in patients with chronic respiratory conditions such as COPD or in obese patients with obesity hypoventilation syndrome [58].

However, NIMV also has limitations. Its effectiveness can be compromised by factors such as patient discomfort, which may lead to poor compliance, and the need for careful monitoring to prevent complications like aspiration or inadequate ventilation. Additionally, NIMV may not be suitable for all surgical patients, particularly those requiring deep sedation or with severe respiratory failure where invasive support might be necessary [59,60].

CPAP is a NIMV strategy that maintains positive airway pressure throughout the respiratory cycle, thereby preventing airway collapse, particularly in patients with OSA or those at risk of PPCs. The mechanism of action involves delivering a continuous flow of air that splints open the upper airway, enhancing oxygenation and reducing the work of breathing [61].

Clinical outcomes associated with CPAP use in the perioperative setting demonstrate a reduction in the incidence of PPCs, including atelectasis and hypoxemia. A randomized controlled trial (RCT) demonstrated that CPAP may reduce the need for endotracheal intubation and other serious complications in patients who experience hypoxemia following elective major abdominal surgery [62]. However, a systematic review and meta-analysis conducted in 2015 found no significant difference in post-operative adverse events between CPAP and no-CPAP treatments. Patients using CPAP had a significantly lower post-operative Apnea-Hypopnea Index and showed a trend toward shorter hospital stays [63].

Bilevel Positive Airway Pressure (BiPAP)

While CPAP delivers a constant pressure through the entire breath cycle, BiPAP delivers a preset inspiratory positive airway pressure and expiratory positive airway pressure [64]. This dual pressure system enhances respiratory support by reducing the work of breathing and improving gas exchange.

Studies indicate that BiPAP can reduce mortality and morbidity rates in patients with severe respiratory conditions by providing effective non-invasive respiratory support. Additionally, it can decrease the need for sedation and shorten the duration of mechanical ventilation [65].

A systematic review and meta-analysis conducted in late 2024 on the efficacy of BiPAP in perioperative care for obese patients undergoing surgery highlights the significant potential of BiPAP therapy in managing these patients during the perioperative period, especially those at high risk for post-operative respiratory complications. BiPAP therapy not only improves oxygenation levels and pulmonary function but also significantly reduces the incidence of atelectasis and shortens hospital stays. These benefits underscore its crucial role in enhancing perioperative outcomes for this patient population [66].

Pressure Support Ventilation (PSV)

PSV is a mode of NIMV that can be used in both NIMV with masks and invasive ventilation with an endotracheal tube, where the ventilator assists the patient's spontaneous breaths by providing a preset level of positive pressure during inspiration. PSV is patient-triggered and pressure-limited, meaning the patient initiates each breath, and the ventilator delivers a set pressure to assist the inspiratory effort. This mode allows the patient to control their own respiratory rate and tidal volume, promoting comfort and synchrony with the ventilator. This support reduces the work of breathing and helps maintain adequate ventilation, particularly in patients with weakened respiratory muscles [67]. PSV is particularly beneficial during the perioperative period for patients with compromised respiratory function, such as those with obesity or COPD. It helps maintain adequate ventilation without the need for invasive procedures, thereby reducing the risk of complications. While PSV is generally well-tolerated, potential complications include patient-ventilator asynchrony and barotrauma if not properly monitored [68,69].

A RCT reported that the incidence of post-operative atelectasis was lower in patients undergoing either laparoscopic colectomy or robot-assisted prostatectomy who received pressure support during emergence from GA compared to those receiving intermittent manual assistance [70].

Oxygenation technique with high-flow nasal oxygen (HFNO) 

HFNO delivers heated and humidified oxygen at high flow rates, exceeding the patient's spontaneous inspiratory flow. This mechanism reduces dead space, improves alveolar ventilation, and generates a mild PEEP, enhancing oxygenation and reducing the work of breathing [71]. HFNO has been shown to enhance oxygenation, reduce the need for reintubation, and shorten ICU stays. It is particularly effective in managing hypoxemic respiratory failure and acute cardiogenic pulmonary edema, as well as in post-operative and post-extubation settings. HFNO serves both as a prophylactic measure against pulmonary complications and as a treatment for ARF, contributing to better post-operative respiratory function [72]. Preoxygenation before endotracheal intubation is beneficial for many patients. Studies have shown that obese ICU patients preoxygenated with HFNO have a significantly reduced risk of severe hypoxemia compared to those using a non-rebreather mask [73]. However, the FLORALI-2 study, which included 313 adult ICU patients with acute hypoxemic respiratory failure, found no significant difference in severe hypoxemia incidence between patients preoxygenated with Non-Invasive Positive Pressure Ventilation and those with HFNO [74]. A recent meta-analysis revealed that HFNO preoxygenation significantly shortened ICU length of stay by an average of 1.8 days. Subgroup analysis indicated that HFNO significantly reduced severe hypoxemia during intubation in patients with mild hypoxemia [75]. The transnasal humidified rapid-insufflation ventilatory exchange (THRIVE) technique, which uses HFNO to maintain oxygenation during intubation, has shown promising results. Studies in children undergoing GA demonstrated that THRIVE significantly prolonged apnea without desaturation compared to jaw support alone [76]. Additionally, the SHINE study found that HFNO improved intubation success rates and reduced desaturation events in neonates [77]. While generally well-tolerated, potential complications include nasal dryness and discomfort. However, with proper humidification and monitoring, HFNO can significantly enhance patient outcomes in the perioperative setting well-tolerated, potential complications include nasal dryness and discomfort. However, with proper humidification and monitoring, HFNO can significantly enhance patient outcomes in the perioperative setting [78].

Comparative analysis of ventilation strategies

Efficacy in Maintaining Oxygenation and Ventilation

When compared to traditional high tidal volume ventilation (10-15 mL/kg), the PLV approach, which uses lower tidal volumes (Vt 6-8 mL/kg) with moderate PEEP, showed superior maintenance of oxygenation levels throughout laparoscopic surgeries [51,79,80]. By preserving functional residual capacity and keeping the alveoli open, the PLV method is linked to improved alveolar stability and helps avoid alveolar collapse (atelectasis) [81]. In laparoscopic procedures, where the Trendelenburg posture and pneumoperitoneum raise the risk of alveolar collapse and oxygenation issues, this increase in ventilation efficiency might be quite important [51]. High tidal volume mechanical ventilation is one of the traditional ventilation techniques used to combat possible hypoxemia. However, alveolar overdistension brought on by an excessive tidal volume has been shown to paradoxically reduce oxygenation by interfering with capillary blood flow in the alveoli [82,83]. In contrast, the PLV strategy helps maintain adequate oxygen levels without imposing strain on alveolar structures [51].

Impact on Intraoperative and Postoperative Outcomes

By emphasizing low tidal volumes with moderate PEEP, PLV reduces lung strain during mechanical ventilation and lowers immediate intraoperative risks such as alveolar hyperexpansion. PLV preserves lung compliance during surgery and lessens the likelihood of alveolar collapse, which leads to more stable respiratory parameters. Following surgery, patients who received PLV had fewer episodes of hypoxemia and could typically maintain oxygen saturation without the need for additional oxygen [79,81].

Conventional ventilation patients, however, had more intraoperative variations in respiratory mechanics and a propensity for alveolar hyperinflation as a result of high tidal volumes. This overdistension frequently resulted in longer recovery periods because of respiratory impairment and higher post-operative oxygen requirements [51]. RCTs such as the IMPROVE trial have underscored some benefits, showing that patients who received PLV strategies experienced reduced incidence of both pulmonary and extrapulmonary complications. These patients also showed improvements in measures such as forced expiratory volume post-surgery, which translated to shorter hospital stays and faster recoveries [79].

Postoperative Pulmonary Complications (PPCs)

PLV was found to significantly lower PPCs, such as respiratory infections, pneumonia, and atelectasis [84]. According to a recent meta-analysis that assesses the impact of protected lung ventilation on pulmonary complications following laparoscopic surgery, PLV reduces the risk of PPCs by about 1.17 times when compared to traditional breathing techniques [51]. Pneumoperitoneum and the Trendelenburg position during laparoscopic procedures frequently worsen atelectasis, the most commonly reported PPC; PLV's use of moderate PEEP (usually 5-10 cm H2O) effectively counteracts these factors by promoting alveolar dilatation and minimizing collapse [81,82,84]. Because of the repeated alveolar overdistension and ensuing inflammatory response, conventional ventilation, with its high tidal volumes, has been associated with an increased incidence of PPCs [51]. These results highlight how PLV can help lower PPCs, especially in surgical contexts where lung integrity is vulnerable to positioning and insufflation pressures.

Risk of Ventilator-Induced Lung Injury (VILI)

PLV techniques greatly reduce ventilator-induced lung damage, a frequent worry in mechanical ventilation. PLV reduces alveolar overstretching, a major contributing cause to VILI development, by utilizing a low tidal volume. PLV lowers the inflammatory mediators that cause VILI and lessens the mechanical stress on lung tissues with small tidal volumes and moderate PEEP [82]. 

On the other hand, higher rates of VILI, defined by damage to the vascular endothelium and alveoli due to persistent alveolar hyperexpansion, were linked to Conventional ventilation with high tidal volumes. This risk not only leads to increased inflammation but also predisposes patients to ARDS if prolonged. PLV, therefore, provides a way to avoid lung damage, which is especially helpful for longer laparoscopic procedures that call for continuous ventilation [51,82].

Hemodynamic Effects

It was shown that PLV had comparatively minor hemodynamic effects because the low PEEP values preserved alveolar patency without causing increases in intrathoracic pressure. More constant blood pressure and cardiac output result from this intrathoracic pressure reduction, which is essential for patient safety during surgery [51]. On the other hand, excessive intrathoracic pressure caused by high PEEP levels, which are frequently used in combination with traditional high tidal volume ventilation, can hinder alveolar hyperexpansion. In turn, pulmonary vascular resistance may rise proportionally, and an imbalance in the ventilatory blood flow ratio may affect hemodynamics and result in PPCs. Therefore, PLV with a moderate PEEP level is a preferable approach for its balance between effective oxygenation and hemodynamic stability [85]. The PROVHILO trial, for example, demonstrated that although high PEEP levels did not significantly reduce PPCs compared to low PEEP in open abdominal surgery, they were associated with more frequent episodes of intraoperative hypotension. This finding underscores the importance of individualized PEEP titration based on each patient's hemodynamic profile to optimize both respiratory and cardiovascular outcomes [86].

Clinical implications and recommendations

The ERS/ATS guidelines recommend NIMV for patients with post-operative ARF [54]. Surgery, especially near the diaphragm, can impair respiratory function, causing hypoxemia and atelectasis. NIMV, including bilevel and CPAP, improves lung aeration and oxygenation, reducing atelectasis without adverse effects. High-flow nasal cannula therapy is also effective, particularly in cardiothoracic surgery patients. Overall, NIV reduces ICU length of stay, tracheal reintubation, and healthcare-associated infections [54].

Limitation of current evidence and future directions

Current evidence on ventilation strategies for patients undergoing GA is limited by small sample sizes, heterogeneity in study designs, and varying patient populations. Future research should focus on large-scale RCTs to establish definitive guidelines. Additionally, exploring the safety and efficacy of conservative oxygen therapy and individualized ventilation strategies could enhance patient outcomes. Emerging technologies such as HFNC therapy, automated closed-loop ventilation systems, and advanced monitoring tools are promising [87]. These innovations aim to optimize ventilation parameters in real-time, improving patient safety and outcomes. Further research should investigate the long-term effects of these emerging technologies, their cost-effectiveness, and their impact on different patient populations. Studies on the integration of artificial intelligence in ventilation management and its potential to predict and prevent complications are also warranted.

## Conclusions

The choice of ventilation strategies during GA is critical for optimizing patient outcomes and minimizing complications. Tailoring these strategies to individual patient characteristics -such as age, comorbidities, and type of surgery- ensures efficacy and safety. The PLV approach, using lower tidal volumes with moderate PEEP, maintains oxygenation and alveolar stability better than traditional methods, reducing hypoxemia, pulmonary complications, and VILI while having minimal hemodynamic effects during surgery. Large-scale RCTs should focus on diverse patient populations and incorporate advanced technologies to refine ventilation management.
